# An in vitro comparison of antimicrobial efficacy and cytotoxicity between povidone-iodine and chlorhexidine for treating clinical endometritis in dairy cows

**DOI:** 10.1371/journal.pone.0271274

**Published:** 2022-07-08

**Authors:** Natcha Thongrueang, Shyh-Shyan Liu, Huan-Yu Hsu, Hsu-Hsun Lee

**Affiliations:** 1 Department of Veterinary Medicine, College of Veterinary Medicine, National Pingtung University of Science and Technology, Neipu, Pingtung, Taiwan; 2 Veterinary Medical Teaching Hospital, Department of Veterinary Medicine, College of Veterinary Medicine, National Pingtung University of Science and Technology, Neipu, Pingtung, Taiwan; 3 Graduate Institute of Animal Vaccine Technology, College of Veterinary Medicine, National Pingtung University of Science and Technology, Neipu, Pingtung, Taiwan; 4 Research Center of Animal Biologics, National Pingtung University of Science and Technology, Neipu, Pingtung, Taiwan; University of Florida, UNITED STATES

## Abstract

This study aimed to assess the in vitro antimicrobial effects of chlorhexidine (CHX) and povidone-iodine (PI) on clinical isolates of *Escherichia coli* (*E*. *coli*) and *Trueperella pyogenes* (*T*. *pyogenes*) from the vaginal discharge of dairy cows, as well as to compare the cytotoxicity effects of CHX and PI on bovine endometrial epithelial cells (BEnEpC). In Experiment 1, 12 *E*. *coli* and 10 *T*. *pyogenes* were isolated from the vaginal discharge of cows with a uterine infection. The MIC and MBC against CHX and PI were analyzed in vitro. In Experiment 2, the cytotoxicity effects of CHX and PI on BEnEpC were analyzed using a Viability/Cytotoxicity Kit, wound scratch healing assay, and the expression of pro-inflammatory cytokine genes (IL-6, IL-8, and TNF-α). In Experiment 1, the MIC and MBC values of CHX against *E*. *coli* were 0.0002% and 0.0002 to 0.00025%, respectively. The MIC and MBC values of PI were 1.25 to 2.5% and 1.25 to 5%, respectively. For *T*. *pyogenes*, the MIC and MBC values of CHX were 0.00002%. The MIC and MBC values of PI were 1.25%. In Experiment 2, the cell viability significantly decreased, and wound closures were significantly inhibited after treatment with ≥ 0.002% CHX and ≥ 0.025% PI. The expression of IL-6, IL-8, and TNF-α significantly increased after treatment with PI. Only IL-6 showed a significant increase after cells were treated with 0.00002% and 0.0002% CHX. The results suggested that both CHX and PI had high antibacterial effects. However, veterinarians and farmers should be aware of their cytotoxicity, which decrease viability of endometrial epithelial cells and inhibit wound healing in vitro.

## Introduction

Clinical endometritis (CE) is an important disease in postpartum cows, as it causes reproductive inefficiency due to lower peripheral plasma estradiol concentrations, the slower growth of the first postpartum dominant follicle, a lower pregnancy rate, and a higher culling rate [1–3]. CE is an inflammation of the endometrial lining of the uterus without systemic signs which is associated with the Gram-positive endometrial pathogenic, *Trueperella pyogenes* (*T*. *pyogenes*); the Gram-negative *Escherichia coli* (*E*. *coli*); and several anaerobes [2, 4]. *E*. *coli* and *T*. *pyogenes* are most frequently isolated from the uterine lumen of postpartum cows and are associated with greater endometrial inflammation and severe clinical uterine diseases [2, 3, 5, 6].

The general principle therapy of CE is to eliminate pathogenic bacteria in the uterus and reverse inflammatory changes that impair fertility [4, 7–9]. A wide variety of therapies for CE have been reported, including systemic prostaglandin F2α (PGF2α) or estradiol, systemic or local antibiotics, and local antiseptics [10–12]. Safe and suitable treatments for CE are needed to avoid problems with residual hormones or antibiotics in milk and meat [13, 14]. Povidone-iodine (PI) solutions are often used for intrauterine infusion for CE owing to their low cost and rapid and highly effective killing of bacteria. Importantly, they do not pass into the milk and meat except in the case of excess administration [15]. However, PI can induce both acute and chronic inflammatory changes in the endometrium in vivo [16]. Chlorhexidine (CHX) is a widely used antimicrobial agent in humans, mares and small animals, but not in dairy cows, as a surgical hand scrub, topical preoperative skin disinfectant, oral rinse, and wound cleanser [17–19]. Nevertheless, adverse reaction of CHX has been observed at concentrations above 0.25% due to severe inflammation on the endometrium of mares [20].

The bovine endometrial epithelium cells (BEnEpC) are the first line of defense in exposure to various invading agents [21]. BEnEpC are often used as a bovine in vitro model for studying the biological characteristics, cellular and molecular mechanisms, development of interactions between the embryo and mother, and responses to infection or damage of the bovine uterine epithelium [22–26]. However, the consequences of antiseptics on BEnEpC have not been described. The studies on cytotoxicity assessments of PI and CHX have generally focused on human cells such as human epithelial HeLa cells, epidermal keratinocytes, dermal fibroblasts and osteoblasts [27–29]. Cell viability analyses in several studies showed that dead cells increased in an application time and concentration‑dependent manner after administration of PI and CHX [17, 30, 31]. The wound scratch healing assay is widely used in different fields of research because it is inexpensive and simple to perform [32]. Human primary osteoblasts, fibroblasts, and myoblasts exposed to ≥ 0.1% PI and ≥ 0.02% CHX showed the scratch defect remained open indefinitely, which may lead to a delay in wound healing [33, 34]. Pro-inflammatory enzymes and cytokines have been suggested to be mediators for inflammatory processes and wound healing, however overproduction may lead to prolonged inflammation and wound recovery [35]. A significantly higher interleukin-1β (IL-1β), IL-6, IL-8 and tumor necrosis factor-α (TNF-α) mRNA content was observed in samples obtained from cows with inflamed endometrium compared with samples from cows with a healthy endometrium [35]. Several studies have estimated cell injury by investigating inflammatory cytokines expression in cultured cells at both mRNA and protein levels [27, 29]. A concentration of 0.0002% CHX can induce human epidermal keratinocytes to secrete IL-1α mRNA expression, but another study showed that human osteoblasts were not induced by 0.1 and 2% CHX to secrete various IL (1β, 6, and 7), interferon γ and TNF-α [27, 29].

The standard clinical treatment by PI for CE in dairy cows is still challenging for veterinarians and farmers in the matter of what concentration can be administered to correct the problems without adverse effects. The current study was designed to assess the antimicrobial effects of CHX and PI on clinical isolates of *E*. *coli* and *T*. *pyogenes* from the vaginal discharge of dairy cows with uterine infection using both their minimum inhibitory concentration (MIC) and minimum bactericidal concentration (MBC). We also aimed to compare the effects of CHX and PI on BEnEpC cell viability, wound healing, and inflammatory cytokine expression using in vitro cell culture techniques.

## Materials and methods

### Experimental 1: Bactericidal effects of CHX and PI in vitro

#### Animals and examinations

This study was conducted in 3 commercial dairy farms in 3 counties (Changhua, Tainan, and Pingtung) in Taiwan. For wild varieties and species of bacteria, farms were selected based on location and farmer participation. All early lactation cows in each farm were examined weekly by a veterinarian to determine their health status and uterine conditions. Transrectal palpation was performed, and the size of uterine horns, fluctuating contents, and vulva discharge was recorded. The vaginal discharge was scored as 0, clear or translucent discharge; 1, discharge containing flecks of white or off-white pus; 2, discharge containing ≤ 50% white or off-white mucopurulent material; 3, discharge containing > 50% purulent material [36]. Cows with a vaginal discharge score (VDS) of more than 0 were enrolled in this study.

#### Collection of vaginal discharge

This study was approved and conducted under the Guiding Principles for the Use and Care of Laboratory Animals and the guidelines of the Institutional Animal Care and Use Committee (IACUC) of the National Pingtung University of Science and Technology (NPUST; approval no. NPUST-IACUC-109-067).

A total of 32 Holstein cows with purulent vaginal discharge (score 3), in a range between 1 and 31 DIM, were enrolled in this study. The vaginal discharge was collected from vulva by pressed uterine and vagina through transrectal palpation. This procedure was performed because the uterine of early lactation cows were always enlarged and accumulated a lot of uterine discharge, that had overflowed to the vagina and vulva. For that reason, the vagina and uterine bacteria were similar [37]. To avoid contamination, the tail of the cow was held, and the vulva and perineum area were disinfected thoroughly by 5% BKC (BENKOCHIO-500, South Asia Chemicals & Feeds Ltd., Taiwan) solution. Vaginal discharge was collected into a 50 mL sterile centrifuge tube and shipped at 4°C for bacterial isolation and molecular detection.

#### Bacterial isolation and molecular detection

Each vaginal discharge sample was cultured on a blood agar plate containing Tryptic soy (TS) agar (BD Difco^™^, Thermo Fisher Scientific Inc., Taiwan) with 5% goat blood and incubated 24–48 hours at 37°C aerobically in a standard microbiology incubator (Fristek Scientific, Taichung, Taiwan). Bacterial species were identified based on their characteristics of the colony, morphology, and gram stain, and individual colonies were collected for future examinations. Colony morphology of *E*. *coli* and *T*. pyogenes were shown in the (S1 Fig) DNA was extracted from selected colonies of *E*. *coli* and *T*. pyogenes, which are the most relevant pathogenic bacterial species in uterine infection, by the boiling method, as described in a previous study [38]. Briefly, the individual bacterial colony was resuspended in 300 μl ultrapure water (Ultrapure water system, UP-DQ plus, Pure Yes Co., Ltd., Taiwan) and incubated for 15 min at 99°C then immediately cooled on ice. The extracted DNA of *E*. *coli* colonies was subjected to the *fimH* gene-specific PCR (508 bp) [39]. The PCR conditions consisted of initial denaturation at 95°C for 12 min, followed by 25 cycles of 94°C for 30 sec, 63°C for 30 sec and 68°C for 3 min. This was followed by a final extension of 10 min at 72°C (S1 Table and S2A Fig). For *T*. pyogenes, the *plo* gene-specific primers were used for DNA amplification reaction (270 bp) [40]. The PCR conditions consisted of initial denaturation at 95°C for 10 min, followed by 35 cycles of 94°C for 1 min, 55°C for 1 min and 72°C for 1 min. This was followed by a final extension of 5 min at 72°C (S1 Table and S2B Fig). The chosen genes, *fimH* gene for *E*. *coli* and *plo* gene of *T*. *pyogenes*, are the most popular genes that have been used for molecular identification and conformation of the isolated bacteria from metritis and endometritis cows [41, 42]. Confirmed bacteria were stored in TS broth (BD Difco^™^, Thermo Fisher Scientific Inc., Taiwan) containing 50% glycerol at -20°C.

#### MIC and MBC of CHX and PI

The standard broth microdilution method (CLSI M07-A9) was used to study the antimicrobial efficacy of CHX and PI by evaluating the growth of bacteria using spectrophotometry. This assay was performed by modifying the method described in previous studies [43, 44]. Briefly, clinical isolates of stored *E*. *coli* and *T*. *pyogenes* were inoculated in 3 ml TS broth and incubated for 24 hours at 37°C. After 24 hours, each solution was diluted with TS broth in order to get 0.5 McFarland bacterial suspension (1×10^8^ CFU/ml). Ten-fold serial dilutions of CHX (0.00002 to 2%) and PI (0.0001 to 10%) were prepared with TS broth in a test tube and mixed with bacterial suspensions to give a final concentration of bacteria approximately 5×10^4^ CFU/ml. A total of 300 μl of each dilution in 3 replicates per dilution was delivered to wells of flat-bottom 96-well microtiter plates with lids to prevent cross-contamination. Control wells were prepared by finial concentration of bacterial suspensions approximately 5×10^4^ CFU/ml. Optical density was determined in a spectrophotometer at 620 nm prior to incubation (T0) and determined again after incubation for 24 hours at 37°C (T24). The MIC was considered as the lowest concentration that inhibited the growth of bacteria after overnight incubation. The MBC was determined by subculturing 100 μl from each negative test well onto TS agar plates and incubating for 24 hours at 37°C. MBC was defined as the lowest concentration resulting in a negative subculture or giving the presence of only one colony after incubation. Two-fold serial dilutions of CHX and PI, which started from the MBC of each bacteria, were prepared to determine the actual MIC and MBC of *E*. *coli* and *T*. *pyogenes*. Each concentration of CHX and PI on each *E*. *coli* and *T*. *pyogenes* was tested in triplicate.

### Experimental 2: Cytotoxicity effects of CHX and PI on BEnEpC

#### Cell culture and treatments

BEnEpC derived from healthy bovine uterus was purchased from Cell Applications, Inc (San Diego, CA, USA). Cells were seeded in standard sterile 75 cm^2^ tissue culture flask and cultured in Dulbecco’s Modified Eagle Medium (DMEM/F12; Gibco, Thermo Fisher Scientific Inc., Taiwan) supplemented with 10% fetal bovine serum (FBS; Corning Incorporated, USA) and Penicillin-Streptomycin-Amphotericin B Solution (Biological Industries, Germany) at 37°C in a humidified atmosphere of 95% air and 5% CO_2_.

Ten-fold serial dilutions of CHX (0.00002 to 2%) and PI (0.00025 to 2.5%) were prepared with ultrapure water. The treatment concentrations were selected based on the MBC of *E*. *coli* and *T*. *pyogenes* in Experiment 1.

#### Cell viability assay

Cell viability assay for BEnEpC was performed using Viability/Cytotoxicity Kit (The LIVE/DEAD^®^ Viability/Cytotoxicity Kit, Thermo Fisher Scientific Inc., Taiwan). The Viability/Cytotoxicity Kit uses a two-color assay to determine the viability of cells in a population based on plasma membrane integrity and esterase activity. Viable cells are simultaneously stained with green-fluorescent calcein-AM to indicate intracellular esterase activity, while dead cells are stained with red-fluorescent ethidium homodimer-1 to indicate loss of plasma membrane integrity. BEnEpC were seeded on sterile microscope coverslips at a density of 8500 cells/cm^2^ and cultured at 37°C in a humidified atmosphere of 95% air and 5% CO_2_ until cells reached about 80 to 90% confluency. Cells were washed with D-PBS (D-PBS; Gibco, Thermo Fisher Scientific Inc., Taiwan) and incubated with serial dilutions of CHX (0.00002 to 2%) and PI (0.00025 to 2.5%) for 1 hour at 37°C in a humidified atmosphere of 95% air and 5% CO_2_ incubator. Negative control (NC; viable cells) was prepared by cells cultured without any treatments, while positive control (PC; dead cells) was prepared by cells cultured with 75% methanol for 30 min. After incubation, BEnEpC were washed with D-PBS twice to remove excess CHX and PI, and then applied Viability/Cytotoxicity Kit according to the manufacturer’s protocol. Two micromolar of calcein AM and 4 μM ethidium homodimer-1 (EtD-1) solution were added to each coverslip and then incubated for 30 min at room temperature. Finally, the coverslip was inverted and mounted on the microscope slide, then visualized under the fluorescence microscope (Olympus BX51 Fluorescence Microscope Cutaway Diagram, Olympus America Inc., New York). Viable cells were stained with green fluorescence by calcein, while EthD-1 entered cells with damaged membranes, produced a bright red fluorescence. Cell viability (%) was calculated as follows: cell viability (%) = {Viable cells / (Viable cells + Dead cells) x 100%}. Each concentration of PI and CHX was tested in triplicate.

#### Wound scratch healing assay

The wound scratch healing assay was used to study the migration ability of the BEnEpC after being exposed to CHX and PI. This assay was performed by a modification of that described in a previous study [34]. BEnEpC were seeded into 24-well plates at a density of 8500 cells/cm^2^ and cultured at 37°C in a humidified atmosphere of 95% air and 5% CO_2_ until cells reached complete confluence. Firstly, the straight line wound in the center of each well was made by a p200 pipette tip. Cells were washed with D-PBS to remove the debris, and then incubated with serial dilutions of CHX (0.00002 to 2%) and PI (0.00025 to 2.5%) for 1 hour at 37°C in a humidified atmosphere of 95% air and 5% CO_2_ incubator. NC and PC were prepared as previously described. After incubation, cells were washed again with D-PBS twice. Finally, cells were cultured with growth media, and images were captured (T0) before being returned to the incubator. Subsequent images at the same field were obtained at 6 (T6), 12 (T12), 24 (T24), 48 (T48) and 72 (T72) hours after incubation. The percentage of areas covered by migrated cells was calculated based on the following: wound closure (%) = (S_T0_-S_TΔ_/S_T0_) x 100%, where S stands for the width of the wound (mm) and T0 is the start time, while TΔ stands for subsequence measured time.

#### RNA extraction and real-time PCR analysis for cytokines measurement

The effects of CHX and PI on the gene expression of cytokines in BEnEpC were evaluated by RT-qPCR. BEnEpC were seeded in 6-well plates at a density of 8500 cells/cm^2^ and cultured at 37°C in a humidified atmosphere of 95% air and 5% CO_2_ until cells reached about 80 to 90% confluency. Cells were washed with D-PBS (D-PBS; Gibco, Thermo Fisher Scientific Inc., Taiwan) and incubated with serial dilutions of CHX (0.00002 to 2%) and PI (0.00025 to 2.5%) for 1, 3, and 5 hours at 37°C in a humidified atmosphere of 95% air and 5% CO_2_ incubator. NC and PC were prepared as previously described. After incubation, cells were washed twice with PBS, and then total RNA was extracted and converted to cDNA using Virus Nucleic Acid Isolation Kit (Cat no.NA016-0100, GeneDireX, Inc., Taiwan) and PrimeScript RT reagent Kit (Takara Bio Inc., Shiga, Japan), respectively. RT-qPCR reactions to quantify mRNA abundance for IL-6, IL-8, TNF-α, and β-actin genes were performed with a CFX Connect Real-Time PCR System (Bio-Rad Laboratories, Inc., California, USA) using an iQ SRBR^®^ Green Supermix (Bio-Rad Laboratories, Inc., California, USA) and the target gene-specific primer sets for bovine (Table 1). The following cycling conditions were performed: 50°C for 2 min, 95°C for 30 sec, 45 cycles of 95°C for 15 sec, 60°C for 30 sec and 72°C 30 sec. All samples were amplified in duplicate, and specific amplification was confirmed by melting-point analysis. Relative mRNA expression levels were determined by normalizing to the expression of a housekeeping gene β-actin, using the 2(-DeltaDeltaC(T)) method (2^-ΔΔCt^).

**Table 1:** Selected gene transcripts, primer sequences and annealing temperatures used for RT-qPCR.

Genes	Forward primers (5’-3’)	Reverse primers (5’-3’)	Annealing temperatures (°C)	References
**IL-6**	AACAGCTATGAACTCCCGCT	GATTTTGTCGACCATGCGCT	91.0	[45]
**IL-8**	CGCTGGACAGCAGAGCTCACA	TGCCAAGAGAGCAACAGCCAGC	81.5	[45]
**TNF-α**	CTCTTCTGCCTGCTGCACTTC	CCATGAGGGCATTGGCATACG	82.0	[45]
**β-actin**	CTAGGCACCAGGGCGTAATG	CCACACGGAGCTCGTTGTAG	86.5	[46]

#### Statistical analysis

For the statistical analysis, SAS (Statistical Analysis System) 9.4 (SAS Institute Inc., North Carolina, USA) software was used. All experiments were conducted in triplicate while the results are presented as mean ± standard error. One-way ANOVA (Kruskal-Wallis’ test) tests followed by a Duncan’s post hoc test were performed to compare cell viability (%) between the NC cells and treated cells. Likewise, wound closure (%) and level of gene expression (IL-6, IL-8, TNF-α and β-actin) between NC and treated cells were compared by the same analysis. Differences were considered statistically significant at p-value < 0.05.

## Results

### Experimental 1: Bactericidal effects of CHX and PI in vitro

#### Samples collection and bacterial isolation

*E*. *coli* and *T*. *pyogenes* were isolated from 50.0% (16/32) and 68.8% (22/32) of vaginal discharge, respectively. Co-infection of *E*. *coli* and *T*. *pyogenes* was found 31.3% (10/32), while these bacteria were not found in 4 samples.

#### MIC and MBC of CHX and PI

A total of 12 clinical isolates of *E*. *coli* from different cows were tested for their MIC and MBC against CHX and PI in vitro. The MIC and MBC values of CHX were found to be 0.0002% and 0.0002 to 0.00025%, respectively. The MIC and MBC values of PI were higher than those of the CHX, 1.25 to 2.5% and 1.25 to 5%, respectively (Table 2). For *T*. *pyogenes*, 10 clinical isolates from different cows were tested. The MIC and MBC values of CHX were found lower than those against *E*. *coli*, 0.00002%. The MIC and MBC values of PI were 1.25% (Table 2).

**Table 2:** MIC and MBC of CHX and PI on clinical isolates of *E*. *coli* and *T*. *pyogenes*.

Bacteria	CHX (%)		PI (%)	
	MIC	MBC	MIC	MBC
***E*. *coli* 1**	0.0002	0.0002	1.25	5
***E*. *coli* 2**	0.0002	0.00025	1.25	2.5
***E*. *coli* 3**	0.0002	0.00025	2.5	2.5
***E*. *coli* 4**	0.0002	0.0002	2.5	2.5
***E*. *coli* 5**	0.0002	0.00025	2.5	2.5
***E*. *coli* 6**	0.0002	0.0002	2.5	2.5
***E*. *coli* 7**	0.0002	0.00025	2.5	2.5
***E*. *coli* 8**	0.0002	0.00025	2.5	2.5
***E*. *coli* 9**	0.0002	0.00025	2.5	2.5
***E*. *coli* 10**	0.0002	0.0002	1.25	2.5
***E*. *coli* 11**	0.0002	0.00025	2.5	2.5
***E*. *coli* 12**	0.0002	0.00025	1.25	2.5
***T*. *pyogenes* 1**	0.00002	0.00002	1.25	1.25
***T*. *pyogenes* 2**	0.00002	0.00002	1.25	1.25
***T*. *pyogenes* 3**	0.00002	0.00002	1.25	1.25
***T*. *pyogenes* 4**	0.00002	0.00002	1.25	1.25
***T*. *pyogenes* 5**	0.00002	0.00002	1.25	1.25
***T*. *pyogenes* 6**	0.00002	0.00002	1.25	1.25
***T*. *pyogenes* 7**	0.00002	0.00002	1.25	1.25
***T*. *pyogenes* 8**	0.00002	0.00002	1.25	1.25
***T*. *pyogenes* 9**	0.00002	0.00002	1.25	1.25
***T*. *pyogenes* 10**	0.00002	0.00002	1.25	1.25

### Experimental 2: Cytotoxicity effects of CHX and PI on BEnEpC

#### Cell viability

**Fig 1:**
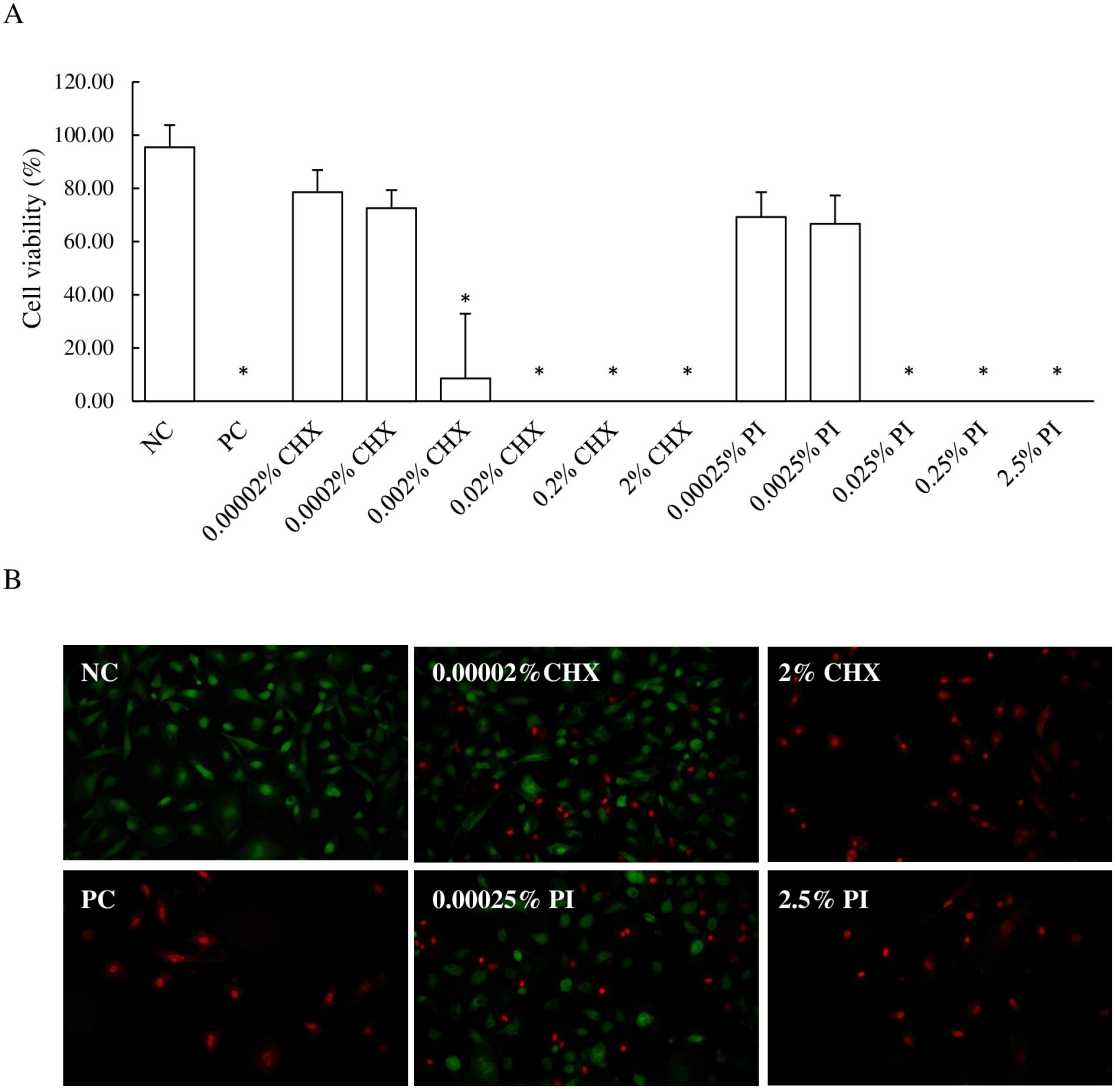
Effect of CHX (0.00002 to 2%) and PI (0.00025 to 2.5%) on BEnEpC cell viability. (A) Percent viability of cells after exposure to CHX and PI for 1 hour at 37°C. The data are means ± SD. *P-value < 0.05 compared to untreated cells (NC). (B) Representative images of the fluorescence analysis (red fluorescence of dead cells and green fluorescence of viable cells). The treatment with 75% methanol (PC) for 30 min, 2% CHX, and 2.5% PI for 1 hour causes cells stained with red-fluorescent, indicating loss of plasma membrane integrity. Cells were stained with green-fluorescent after being exposed with 0.00002% CHX and 0.00025% PI for 1 hour indicated intracellular esterase activity.

The viability of BEnEpC was dose-dependent declined with the increasing of CHX and PI concentrations (Fig 1A). Cells that were treated with 0.00002% and 0.0002% CHX remined viable (78.49±8.39% and 72.55±6.70%, respectively), while %viable cells decreased significantly after being treated with higher concentrations (0.002 to 2% CHX) when compared with NC (95.44±8.33). For PI treatments, cells that were treated with 0.00025% and 0.0025% PI were still viable (69.19±9.31 and 66.66±10.66, respectively), whereas viable cells were significantly decreased when treated with the other concentrations. As seen in Fig 1B, most BEnEpC were stained with green-fluorescent calcein-AM when unexposed to any treatments (NC), while stained with red-fluorescent ethidium homodimer-1 when exposed to 75% methanol for 30 min (PC). Cells that were exposed to 0.00002% CHX and 0.00025% PI were stained with both green and red-fluorescent.

#### The effects of CHX and PI on wound healing

**Fig 2:**
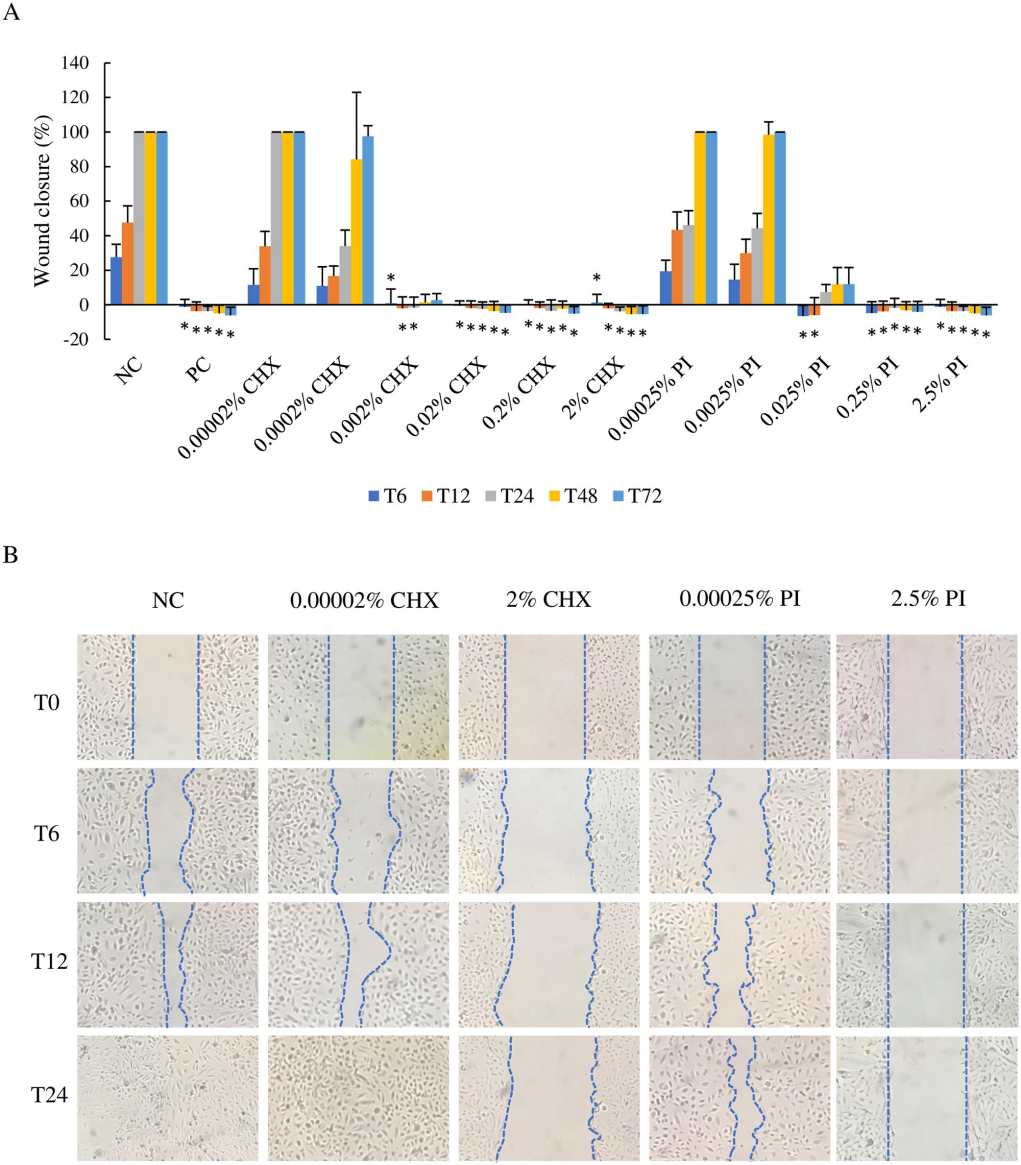
Effect of CHX (0.00002 to 2%) and PI (0.00025 to 2.5%) on BEnEpC wound closure. (A) Percent wound closure of cells at T6, T12, T24, T48, and T72 after exposure to CHX and PI for 1 hour at 37°C. The data are means ± SD. *P-value < 0.05 compared to untreated cells (NC) at each time point. (B) Wound closure pictures of cell monolayers at T0, T6, T12, and T24. Cellular closure of defect was observed within T24 in cells exposed to 0.00002% CHX and no treatment cells (NC). Delay wound defect closure was seen in 0.00025% PI. Cells that were exposed to 2% CHX and 2.5% PI failed to close the scratch defect until the end of the experiment.

The wound scratch healing assay was performed to determine both migration and wound healing ability of BEnEpC after being exposed to CHX and PI for 1, 3, and 5 hours (Fig 2A and 2B). The difference in wound closures size between NC and treated cells at each time point was analyzed. The results showed that the size of wound closure was significantly inhibited after being exposed to 75% methanol (PC), 0.02 to 2% CHX, and 0.25 to 2.5% PI at each time point (p < 0.05). Additionally, their wound closures were not complete until the end of the experiment (T72). For the lower concentrations, including 0.002% CHX and 0.025% PI, wound closures were also significantly inhibited at the first phase; however, slightly increasing wound closures were found at T48 (1.33±4.78) and T24 (7.34±4.36), respectively. The concentration of 0.00002% CHX, 0.0002% CHX, 0.00025% PI, and 0.0025% PI had no significant impact on the wound closure at each time point. Their wound closures were complete within T72, excluding 0.0002% CHX that the failure was found (97.48±6.18%).

#### Effects of CHX and PI on cytokines gene expression in BEnEpC

**Fig 3:**
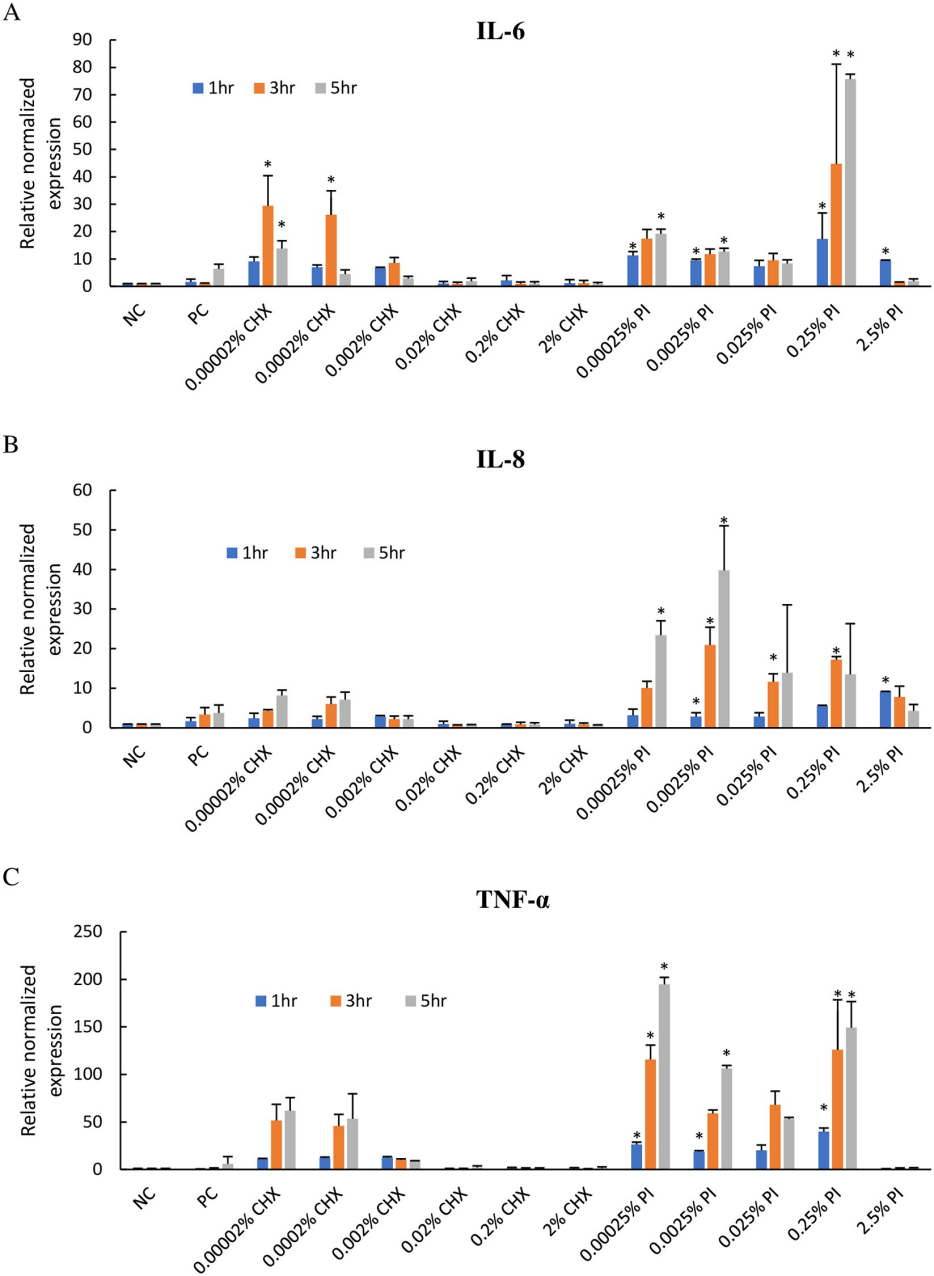
The mRNA expression of inflammatory cytokine. (A) IL-6, (B) IL-8, and (C) TNF-α in BEnEpC treated with CHX and PI for 1, 3, and 5 hours. The mRNA expression levels determined by the quantitative real-time RT-PCR were normalized to β-actin and plotted relative to those of untreated cells (NC). The data were shown as mean ± SD. *P-value < 0.05 compared to untreated cells (NC).

The expression of inflammatory cytokine genes on BEnEpC treated with CHX and PI for 1 hour were evaluated as shown in Fig 3A–3C. IL-6, IL-8, and TNF-α are widely used in in vitro experiments as biomarkers for stimulus responses. The results of RT-qPCR analysis indicated that IL-6 mRNA expression significantly increased in cells treated with CHX concentration 0.00002% and 0.0002% at T3 (p < 0.05) and drastically decreased at T5, while IL-8 and TNF-α mRNA expressions continually increased at T1 to T5 but statistical significance was not shown when compared with NC cells (p > 0.05). The cells that were treated with 0.002% CHX showed a slight increase in IL-6, IL-8, and TNF-α at T1 and decreased over time. The rest concentration of CHX (0.02% to 2%) had no significant impact on the IL-6, IL-8, and TNF-α mRNA expressions.

PI seems to have a higher effect on inflammatory cytokine genes expression. For the cells that were treated with 0.00025% to 0.25% PI, the expression of IL-6, IL-8, and TNF-α were significantly higher than that in NC (p < 0.05) except 0.025% PI, where only the expression of IL-8 was significantly higher than that in NC (p < 0.05). For 2.5% PI treated cells, IL-6 and IL-8 mRNA expressions significantly increased at T1 and decreased over time; however, it had no impact on TNF-α mRNA expression.

## Discussion

In the present study, the effects of commercial antiseptics, including CHX and PI, have been investigated in vitro on field strains of pathogenic bacteria from cows with uterine infection and BEnEpC. The bacteriologic analyses of this study were focused on the identification of *E*. *coli* and *T*. *pyogenes*, which are categorized as recognized uterine pathogens [5, 6]. The prevalence of *T*. *pyogenes* in this study agrees with previous reports, ranging from 65.3 to 81.3%, but *E*. *coli* prevalence was found to be higher (6.3 to 35.6%) [47, 48]. The sampling period of studies could be the main reason for these different findings. The samples in this study were collected from cows between 1 and 31 days postpartum, while other studies were collected from cows more than 21 days postpartum. A previous study found that *E*. *coli* was more frequently isolated in the first few days after calving and decreased during the postpartum period, whereas *T*. *pyogenes* was identified more often later [3, 5]. Thus, our results should support the hypothesis that early infection with *E*. *coli* leads to further infection with *T*. *pyogenes* in the later postpartum period [5].

PI is a broad-spectrum antiseptic against both Gram-positive and Gram-negative bacteria, while CHX is a broad-spectrum antiseptic against Gram-positive bacteria but a narrow-spectrum against Gram-negative bacteria [49]. Both of them have been used to treat uterine infection and improve the fertility of cows and mares [7–9, 48, 50, 51]. However, there have been few studies to determine the efficacy and safety of these compounds. To our best knowledge, this is the first study to determine MIC and MBC of CHX and PI against field strains *E*. *coli* and *T*. *pyogenes* isolated from vaginal discharge of cows with uterine infection. Our study demonstrated that MBC of CHX was in concentrations of 0.0002 to 0.00025% against *E*. *coli* and 0.00002% against *T*. *pyogenes*, while MBC of PI was in concentrations of 1.25 to 5% and 1.25% against *E*. *coli* and *T*. *pyogenes*. These findings were in contrast with previous studies that reported the MBC of CHX was in concentration of 0.1% against *E*. *coli* [8], and another study reported that both 0.5% and 2% PI had high antiseptic effects by inhibiting more than 99.9% of bacterial growth against *T*. *pyogenes* isolated from endometrial membranes of normal calving cows [48]. This variation might be due to the difference between bacterial inoculant strains and the inoculation method. The worldwide data showed that there is increasing resistance among field isolated bacteria to antiseptics; however, there has been no report of bacteria from cows with uterine infection being resistant to antiseptics, whether CHX or PI [52, 53]. To avoid the possibility of inaccurate results, this study collected various field strains of *E*. *coli* and *T*. *pyogenes* from different cows in different farms to determine the exact MIC and MBC values. Moreover, the inoculation method in this study was determined by the standard methods of the Clinical and Laboratory Standards Institute (CLSI), which should minimize any deviations that might occur [44].

The endometrial epithelial cells are the first line of a physical defense mechanism against microbial attachment and invasion during the postpartum period [21]. The endometrial epithelial cells also act as immune sensors, immune effectors and regulators of immune cells [54]. Intrauterine infusion in healthy mares by 1% PI showed significant increases in the numbers of inflammatory cells in the endometrium. Thereafter, the inflammatory reaction changed in nature from an acute to a more chronic reaction [16]. On the other hand, intrauterine infusion by 2% PI in healthy cows induced transient uterine inflammation, promoted regeneration of endometrial epithelial cells, and improved fertility [9]. In human research, many types of cells are well studied on cytotoxicity caused by CHX and PI solutions [16, 31, 34, 55–57]. However, there is a lack of knowledge of the effects of CHX and PI on endometrial epithelial cells in cattle. This study demonstrated that 0.002% CHX and 0.025% PI exposure to BEnEpC for 1 hour significantly reduced cell viability, as well as halted cell migration for at least 72 hours in a concentration-dependent manner. The cytotoxicity of CHX and PI dilutions on fibroblasts, myoblasts, and osteoblasts also are similarly specific concentration-dependent [33, 34].

Interestingly, the cells that were exposed to 0.002% CHX and 0.025% PI in this study demonstrated a significant reduction in cell viability at T0; however, the cell migration was still observed at T48 and T24, respectively. This finding was in contrast to previous studies that showed cell migration has always been in accordance with cell viability, as measured via the scratch test and Cell Counting Kit-8 (CCK-8) [33, 34]. This discrepancy may be because our study assessed cell death using fluorometric assay (calcein-AM and ethidium homodimer-1) followed by manual cell count under a fluorescence microscope, which may be less sensitive for quantifying very small amounts of the viable cells than CCK-8, a colorimetric assay which uses a microplate reader to detect metabolically-active cells [58].

Many studies have reported that cytokines are involved in inflammatory reactions caused by cell damage due to antiseptics. One study showed that IL-1α mRNA expression was evaluated in human epidermal keratinocytes treated with octenidine dihydrochloride, benzalkonium chloride, 3PHBO-12, and CHX [29]. Another study showed polyhexanide, but not CHX, induced human osteoblasts to secrete various IL (1β, 6, and 7), interferon γ, and TNF-α [27]. In this study, the mRNA expression of pro-inflammatory cytokines, including IL-6, IL-8, and TNF-α, served as a biomarker for cell damage and inflammation. Our study demonstrated that the high concentration of CHX (0.002 to 2%) had no significant impact on the IL-6, IL-8, and TNF-α mRNA expression, but the low concentrations of CHX (0.00002 and 0.0002%) did. These were consistent with the previous study, in which 0.1 and 2% CHX treated human osteoblast as short as 1 or 10 minutes could not induce any cytokine (IL-1β, IL-6, IL-7, IFN-γ, TNF-α) to release [27]. Additionally, even when human epidermal keratinocytes were treated with 0.002% CHX for 72 h, IL-11α mRNA expression was unchanged [29]. The unaltered mRNA expression of all cytokines found in this study could be due to the fact that CHX induced cell dysfunction and interfered mRNA expression of BEnEpC. The previous study demonstrated that CHX exerted an inhibitory effect on DNA synthesis from concentrations as low as 0.0001% and reached a maximum at a concentration of 0.02% [59]. CHX also inhibited amino acid incorporation into protein-like material at levels at or above 0.004% [60]. On the contrary, increased cytokines mRNA expression at a low concentration of CHX treated cells could confirm the cytotoxicity of CHX from concentrations as low as 0.00002%.

To the best of our knowledge, the current study is the first report to investigate the effect of PI on pro-inflammatory cytokines mRNA expression by BEnEpC. Generally, PI showed the ability of non-specifically induced cytotoxicity due to the oxidizing effects of free iodine on the functional groups of amino acids, nucleotides and the double bonds of unsaturated fatty acids [61]. Over the last years, PI has been shown to induce cell death through necrosis or apoptosis along with the high production of reactive oxygen intermediates and low mitochondrial membrane permeability [28, 62, 63]. Recently, in contrast, data suggested that cellular fixation was the primary mechanism of cell death from PI rather than necrosis or apoptosis [64, 65]. In this study, the significant increase in inflammatory cytokines mRNA expression after being incubated into 0.00025 to 2.5% PI for 1, 3, and 5 hours seem to be contrary to the concept of cellular fixation, in which the fixed cell could neither respond to external stimuli nor release inflammatory cytokines [64, 65]. The discrepancy between our findings and previous reports may be explained by the method for measurement of cytokines. The previous study assessed cytokine using a Sandwich ELISA kit, which detected secreted cytokine protein in the medium, while our study assessed using RT-qPCR, a highly sensitive method that detected cytokine mRNA [65]. As to the fact that not all mRNA signals are translated to proteins, the previous studies suggested analyzing cytokine mRNA profiles by RT-qPCR, which is reliable in detecting a very low abundance of biological molecules, with a wider detection span, and less affected by post-sampling handling [66, 67]. Therefore, in summary, we were able to clearly demonstrate the highly antibacterial activity of both CHX and PI against the recognized bovine uterine pathogens *E*. *coli* and *T*. *pyogenes*. CHX at a concentration ≥ 0.00025% and PI at ≥ 2.5% inhibited more than 99.9% of bacterial growth. CHX seemed to have superiority when compared with the use of PI in vitro in the matter of lower cell cytotoxicity; however, due to the fact that CHX is pH-dependent and is greatly reduced in the presence of organic matter, especially purulent or mucopurulent discharge, it is the main organic matter in the uterine of cows that present CE [49, 68]. Future studies are required to investigate the in vivo responses of CE cows and the reproductive performance following treatment with CHX and PI.

## Conclusion

The concentrations of ≥ 0.00025% CHX and ≥ 2.5% PI showed high antibacterial effects against pathogenic bacteria isolated from the vaginal discharge of uterine infected cows. However, veterinarians and farmers should be aware of the potential adverse effects of CHX and PI due to their cytotoxicity, which decrease viability of endometrial epithelial cells and inhibit wound healing in vitro.

## Supporting information

S1 Fig Colony morphology of (A) *E*. *coli* and (B) *T*. *pyogenes* on a blood agar plate after incubated 24–48 hours at 37°C. (TIF)

S2 Fig Representative results of PCR amplification of genomic DNA of vaginal discharges. by PCR. (A) *E*. *coli* (508 bp) Land1: 100 bp DNA ladder, Land2: positive control, Land3 positive samples, Land4: negative control. (B) *T*. *pyogenes* (270 bp) Land1: 100 bp DNA ladder, Land2: negative control, Land3 positive control, Land4-8: positive samples.
(TIF)

S1 Table Primer pairs used to amplify each target gene. (DOCX)
