# Supplementary figures and images for: An in vitro comparison of antimicrobial efficacy and cytotoxicity between povidone-iodine and chlorhexidine for treating clinical endometritis in dairy cows
Source: PLoS One. 2022 Jul 8;17(7):e0271274. doi: 10.1371/journal.pone.0271274 (PMC9269917; doi:10.1371/journal.pone.0271274)

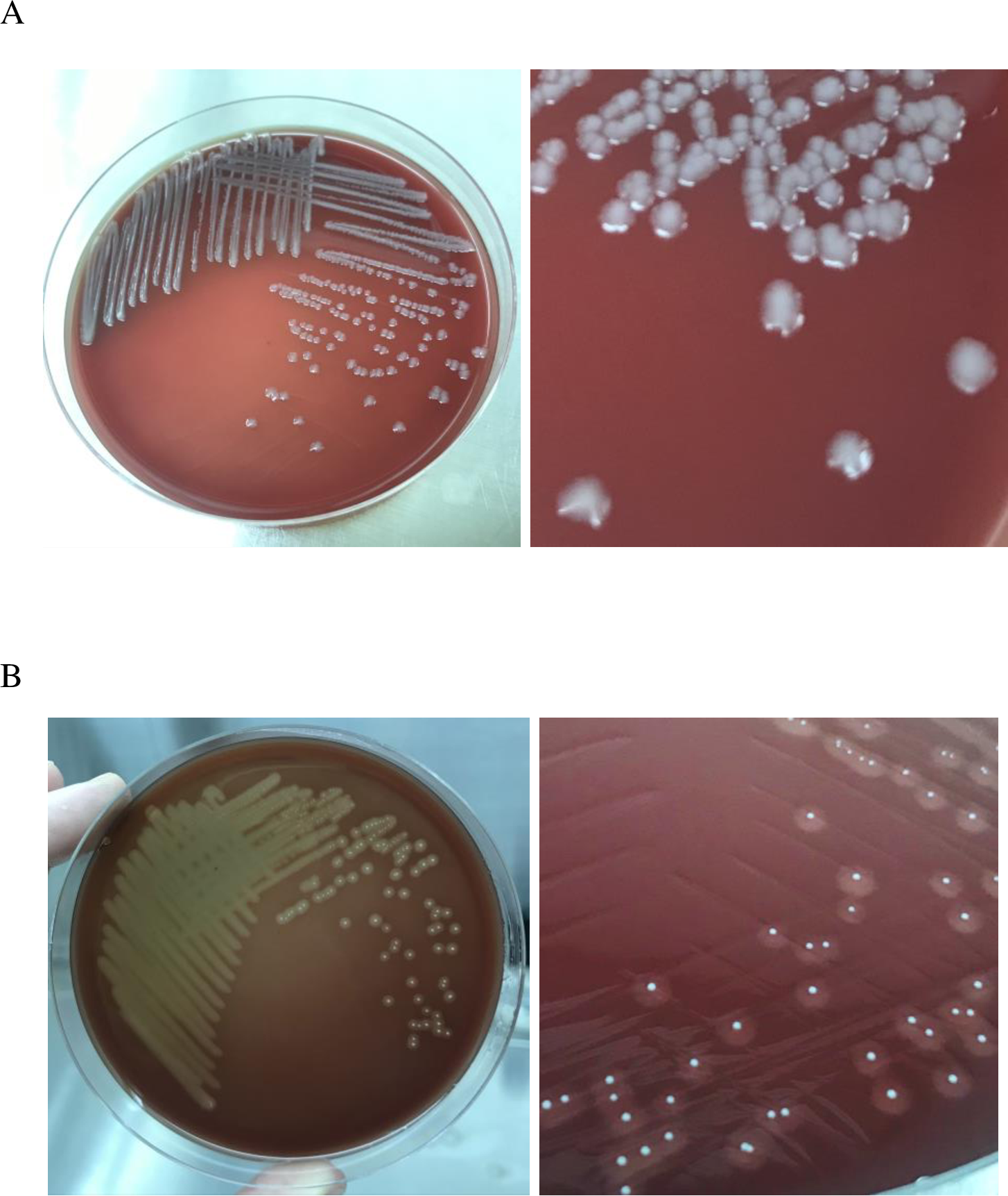

Supplement: S1 Fig — (TIF) [file pone.0271274.s001.tif]

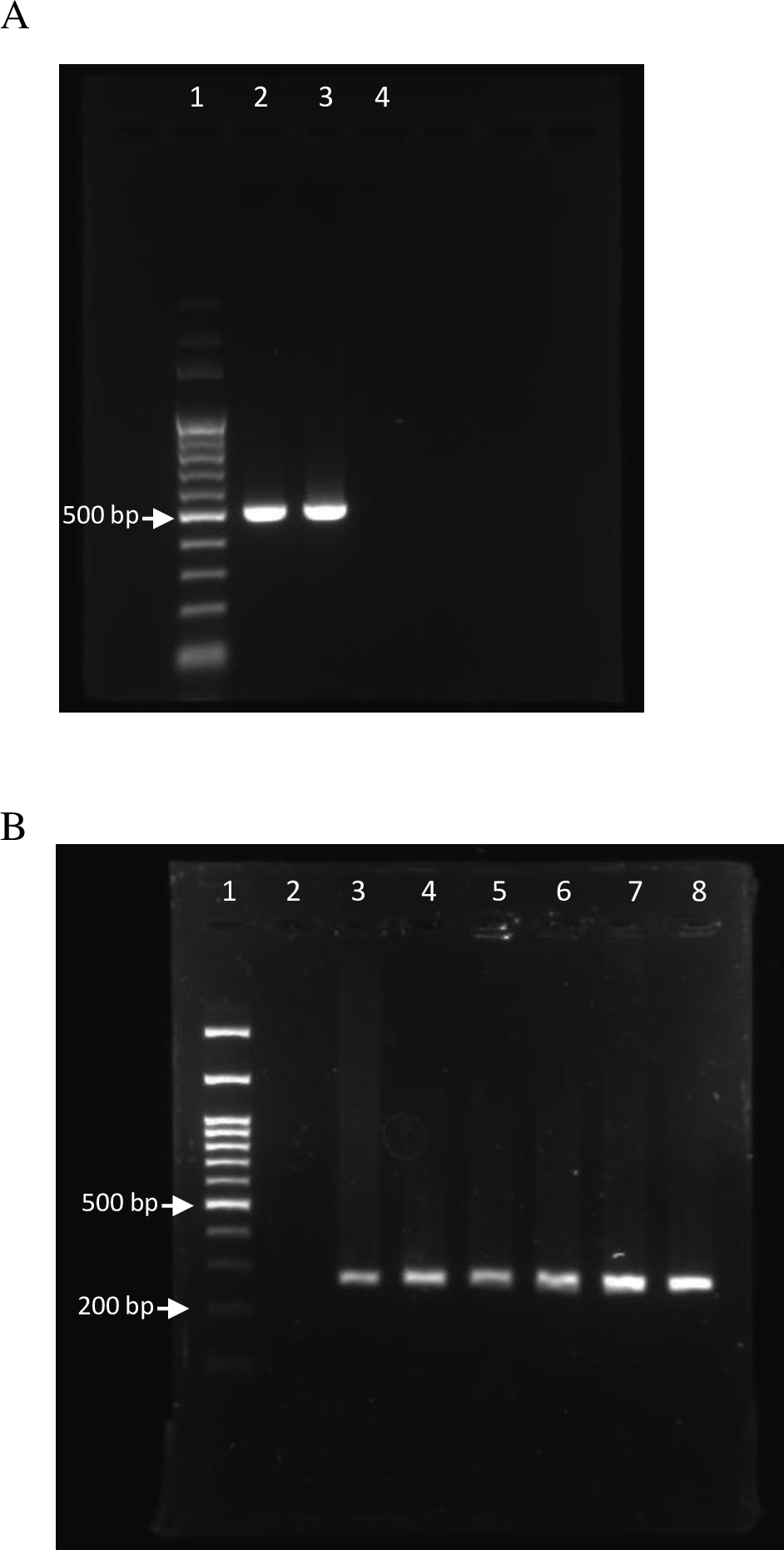

Supplement: S2 Fig — (A) E. coli (508 bp) Land1: 100 bp DNA ladder, Land2: positive control, Land3 positive samples, Land4: negative control. (B) T. pyogenes (270 bp) Land1: 100 bp DNA ladder, Land2: negative control, Land3 positive control, Land4-8: positive samples. (TIF) [file pone.0271274.s002.tif]
