# Supplementary material for: An in vitro comparison of antimicrobial efficacy and cytotoxicity between povidone-iodine and chlorhexidine for treating clinical endometritis in dairy cows
Source: PLoS One. 2022 Jul 8;17(7):e0271274. doi: 10.1371/journal.pone.0271274 (PMC9269917; doi:10.1371/journal.pone.0271274)
Supplement: S1 Table — (DOCX) [file pone.0271274.s003.docx]

**Table S1: Primer pairs used to amplify each target gene.**

| **Species** | **Genes** | **Forward primers (5’-3’)** | **Reverse primers (5’-3’)** | **Product** |
| --- | --- | --- | --- | --- |
| ***E. coli*** | *fimH* | TGCAGAACGGATAAGCCGTGG | GCAGTCACCTGCCCTCCGGTA | 508 |
| ***T. pyogenes*** | *plo*-Pyolysin | GGCCCGAATGTCACCGC | AACTCCGCCTCTAGCGC | 270 |
